# P2Y11 Agonism Prevents Hypoxia/Reoxygenation- and Angiotensin II-Induced Vascular Dysfunction and Intimal Hyperplasia Development

**DOI:** 10.3390/ijms22020855

**Published:** 2021-01-16

**Authors:** Marie Piollet, Adrian Sturza, Stéphanie Chadet, Claudie Gabillard-Lefort, Lauriane Benoist, Danina-Mirela Muntean, Oana-Maria Aburel, Denis Angoulvant, Fabrice Ivanes

**Affiliations:** 1 EA4245 Transplantation, Immunology and Inflammation Laboratory, Loire Valley Cardiovascular Collaboration, University of Tours, 37000 Tours, France; marie.piollet@gmail.com (M.P.); stephanie.chadet@univ-tours.fr (S.C.); claudie.gabillard@gmail.com (C.G.-L.); benoistlauriane@gmail.com (L.B.); denis.angoulvant@univ-tours.fr (D.A.); 2 Department of Functional Sciences-Pathophysiology, Center for Translational Research and Systems Medicine, “Victor Babeș” University of Medicine and Pharmacy Timișoara, 300041 Timișoara, Romania; sturza.adrian@umft.ro (A.S.); daninamuntean@umft.ro (D.-M.M.); oanaduicu@umft.ro (O.-M.A.); 3 Department of Cardiology, Tours University Hospital, 37000 Tours, France

**Keywords:** cardiovascular protection, P2Y purinoreceptor, vascular dysfunction, ischemia reperfusion injury

## Abstract

Vascular dysfunction in cardiovascular diseases includes vasomotor response impairments, endothelial cells (ECs) activation, and smooth muscle cells (SMCs) proliferation and migration to the intima. This results in intimal hyperplasia and vessel failure. We previously reported that activation of the P2Y11 receptor (P2Y11R) in human dendritic cells, cardiofibroblasts and cardiomyocytes was protective against hypoxia/reoxygenation (HR) lesions. In this study, we investigated the role of P2Y11R signaling in vascular dysfunction. P2Y11R activity was modulated using its pharmacological agonist NF546 and antagonist NF340. Rat aortic rings were exposed to angiotensin II (AngII) and evaluated for their vasomotor response. The P2Y11R agonist NF546 reduced AngII-induced vascular dysfunction by promoting EC-dependent vasorelaxation, through an increased nitric oxide (NO) bioavailability and reduced AngII-induced H_2_O_2_ release; these effects were prevented by the use of the P2Y11R antagonist NF340. Human vascular SMCs and ECs were subjected to AngII or H/R simulation in vitro. P2Y11R agonist modulated vasoactive factors in human ECs, that is, endothelial nitric oxide synthase (eNOS) and endothelin-1, reduced SMC proliferation and prevented the switch towards a synthetic phenotype. H/R and AngII increased ECs secretome-induced SMC proliferation, an effect prevented by P2Y11R activation. Thus, our data suggest that P2Y11R activation may protect blood vessels from HR-/AngII-induced injury and reduce vascular dysfunctions. These results open the way for new vasculoprotective interventions.

## 1. Introduction

The renin–angiotensin-aldosterone system (RAAS) plays a major role in cardiovascular physiology and physiopathology and is implicated in endothelial function, vascular remodeling and inflammation [1,2]. It is activated in most cardiovascular diseases, including arterial hypertension, heart failure and ischemic heart disease. Among all the actors of the RAAS, angII is of major importance. Similarly to systemic hypertension, ischemia/reperfusion (I/R) injuries are associated with increased AngII concentration and expression of its specific receptor, the AngII type 1 receptor (AT1) [3]. AT1 is widely expressed and mediates most AngII-related effects such as vasoconstriction, SMC proliferation, fibrosis and inflammation. These effects contribute to increased blood pressure, potentially damaging blood vessels [4]. Hence, blocking this receptor decreased the induced-vascular injury in a model of AngII-induced cardiac vasculopathy, by decreasing tissue factor expression [5].

Ischemia/reperfusion is a major pathophysiological phenomenon in cardiovascular diseases. Whether it is a result of athero-thrombosis, cardiovascular surgery or heart failure, first line consequences are observed in the vasculature of various organs, for example, myocardium, kidney or brain. Ischemia/reperfusion (I/R) induces vascular injuries and imbalanced vascular responses, leading to endothelial dysfunction. I/R conditioning studies for cardioprotection generally focused on beneficial effects of cardiomyocytes, thus forsaking coronary vasculature. However, these conditioning therapies may also affect blood vessels, and therefore participate in the whole organ protection [6]. Among these conditioning interventions, ischemic post-conditioning has been demonstrated to decrease infarct size, tissue edema, P-selectin expression in coronary vascular endothelium and to improve vasodilation response [7]. It also improved endothelial function and reduced no-reflow and necrosis area in a mini-swine model of coronary occlusion [8]. In a mouse model of acute myocardial infarction, preservation of vascular integrity by angiopoietin-like 4 was shown to be protective against I/R injuries and no-reflow [9]. Thus, targeting vascular injuries through pharmacological post-conditioning showed encouraging beneficial results, and may represent new therapeutic insights to prevent vascular injuries and dysfunction, and subsequent organ damages.

Injured cells release extracellular adenosine triphosphate (eATP), which is recognized as a damage associated molecular pattern. It can affect vascular cells, through the modulation of EC apoptosis and activation but also smooth muscle cells (SMC) proliferation. eATP activates specific purinergic receptors, such as ionotropic P2X receptors and some members of the metabotropic P2Y receptor family. Liu and collaborators showed that eATP induced vasodilation in rat small mesenteric arteries by acting on both P2X7 and P2Y11 in ECs [10]. In a rat model of LPS-induced acute systemic inflammation and vascular dysfunction, the activation of a P2Y11-like receptor by the specific agonist NF546 restored basal vasomotor function [11]. This P2Y11 agonist previously demonstrated protective effects against I/R injuries in human cardiomyocytes [12], human cardiofibroblasts [13], and in a rodent model of heterotopic cardiac allograft [14]. It also induced immunomodulatory effects on human dendritic cells in response to a pro-inflammatory hypoxia/reoxygenation sequence [15], as well as beneficial effect in circulatory shock in a model of mouse cardiomyocytes [16]. Therefore, it appears important to investigate the role of P2Y11R on vascular I/R injuries.

This study aimed at assessing the role of P2Y11 modulation on vascular dysfunction in two models of intrigued stress conditions: high concentrations of AngII and (H/R) injury. We hypothesized that P2Y11 activation could reduce vascular dysfunction and mediate SMC and EC cross-talk after injuries, limiting intimal hyperplasia and endothelial dysfunction.

## 2. Results

### 2.1. The P2Y11 Agonist NF546 Improves Angiotensin II—Induced Vascular Dysfunction

**Figure 1:**
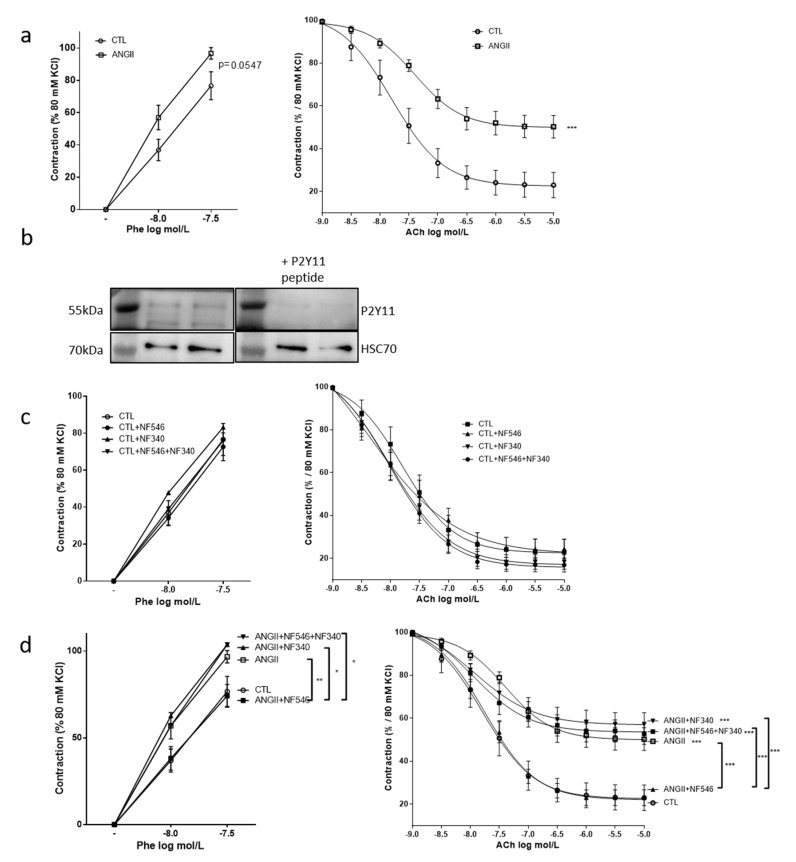
The P2Y11 agonist NF546 restores vascular function impaired by angiotensin II stimulation. Rat aortic rings were incubated 30min with or without angiotensin II (AngII) (100 nM), NF546 (10 µM), or NF340 (10 µM), then vasomotor response was evaluated in response to increasing concentrations of phenylephrine (Phe) (1.10^−8^ M–5.10^−7^ M) and acetylcholine (Ach) (1.10^−9^ M–1.10^−5^M). (**a**) AngII tended to increase the vasoconstrictive response to Phe (*p* = 0.0547) and significantly reduced (*p* < 0.001,) vasorelaxation in response to Ach compared to the control (CTL) (n = 12). (**b**) Expression of a P2Y11-like protein was analyzed by western blot on aortic rings lysate. A band at 55 and 35 kDa could be immunodetected using anti-hP2Y11 primary antibody, and this was prevented by the use of hP2Y11 peptide (n = 2). (**c**) In the CTL group, NF546 or NF340 P2Y11 ligands did not show any significant effects on vascular tone (n = 12). (**d**) In the AngII-treated group, the P2Y11 agonist NF546 significantly restored basal vasomotor response, while the P2Y11 antagonist NF340 had no effect when used alone, but abolished the effect of NF546 when co-applied (n = 12). * *p* < 0.05, ** *p* < 0.01 and *** *p* < 0.001, compared to CTL condition if not indicated.

Aortic rings were incubated with AngII to mimic an environmental stress and to induce a vascular dysfunction. Incubation with AngII showed a tendency to increase vascular constriction in response to phenylephrine (*p* = 0.05) and significantly impaired relaxation in response to acetylcholine as compared to the control (CTL) group (Figure 1a). While the expression of a P2Y11-like receptor is questioned in rodent, expression of a P2Y11-like receptor in rat thoracic aortas was evaluated and confirmed by western blot (Figure 1b). In the CTL group, neither the P2Y11 agonist NF546 nor the P2Y11 antagonist NF340 modified vasomotricity (Figure 1c). However, in aortic rings stimulated with AngII, the application of the P2Y11 agonist NF546 significantly restored vascular functions by reducing vascular contraction and increasing vascular relaxation responses (Figure 1d). Incubation with the P2Y11 antagonist NF340 had no significant effect in the AngII-treated group. Co-incubation with P2Y11 agonist NF546 and antagonist NF340 did not significantly modify vascular function in the AngII-treated group (Figure 1d). These data strongly suggest that the pharmacological activation of a P2Y11-like receptor may improve vascular response during AngII-induced vascular dysfunction, thus restoring the balance of vasomotor factors. 

### 2.2. Beneficial Effect of P2Y11 Agonist Is Dependent on Endothelial Cells

**Figure 2:**
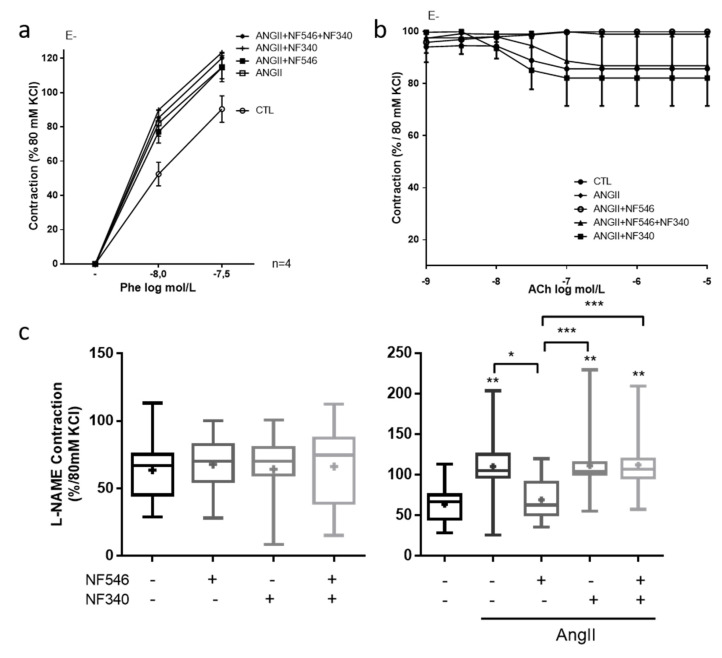
P2Y11 ligands regulate vascular tone in an endothelial cell (EC)-dependent manner and through the modulation of nitric oxide (NO) bioavailability. EC-denuded aortic rings (E-) were incubated for 30 min with or without AngII (100 nM), NF546 (10 µM), or NF340 (10 µM), then vasomotor response was evaluated in response to increasing concentrations of phenylephrine (Phe) (1.10^−8^ M–5.10^−7^ M) and acetylcholine (Ach) (1.10^−9^ M–1.10^−5^M). (**a**) AngII incubation tended to increase the vasoconstrictive response to Phe. The P2Y11 agonist NF546 did not modulate vasoconstriction in AngII-treated groups (n = 4). (**b**) We observed no relaxation in response to Ach after EC denudation (n = 4). (**c**) Rat aortic rings were incubated 30 min with or without AngII (100 nM), NF546 (10 µM), or NF340 (10 µM), and preconstricted with Phe to reach 10% of the KCl maximal constriction level. Response to L-NAME (10 µM) was then measured. In the CTL group, P2Y11 ligands did not show any significant difference on L-NAME response. Contraction elicited by L-NAME was significantly increased in the presence of AngII incubation with NF546 preventing the AngII pro-constrictor effect, while NF340 had no effect (n = 13). Co-administration of both NF546 and NF340 did not prevent the contraction induced by AngII. * *p* < 0.05, ** *p* < 0.01 and *** *p* < 0.01, compared to CTL condition if not indicated.

To assess whether the effect of P2Y11 ligands targeted EC, SMC or both cell types, we performed similar experiments on aortic rings denuded from endothelium. After EC removal, AngII still tended to increase the constrictive response to phenylephrine compared to CTL (Figure 2a), as observed in the presence of endothelium (Figure 1a). However, acetylcholine induced no relaxation (Figure 2b). Furthermore, the incubation with either NF546 or NF340 or both had no effect (Figure 2a), suggesting that the beneficial effect of P2Y11 agonist on vascular function is dependent on EC. 

### 2.3. P2Y11 Agonist Improves Nitric Oxide Bioavailability 

To assess the effect of P2Y11 ligands on nitric oxide bioavailability, we measured the vascular contractility in response to the eNOS inhibitor L-NAME. P2Y11 ligands demonstrated no significant effect in the CTL group (Figure 2c). AngII incubation significantly increased contraction in response to L-NAME compared to the CTL (from 63.6% ± 6.5 to 110.3% ± 12.3 of contraction) and NF546 significantly reduced the constrictive response to L-NAME (from 110.3 ± 12.3 to 69.3 ± 12.3) (Figure 2c). These results suggest that P2Y11 agonists can improve endothelium-dependent relaxation after AngII treatment by increasing NO bioavailability. 

### 2.4. Vascular Tone Regulation by EC Is Modulated by P2Y11R in Favor of Relaxation

**Figure 3:**
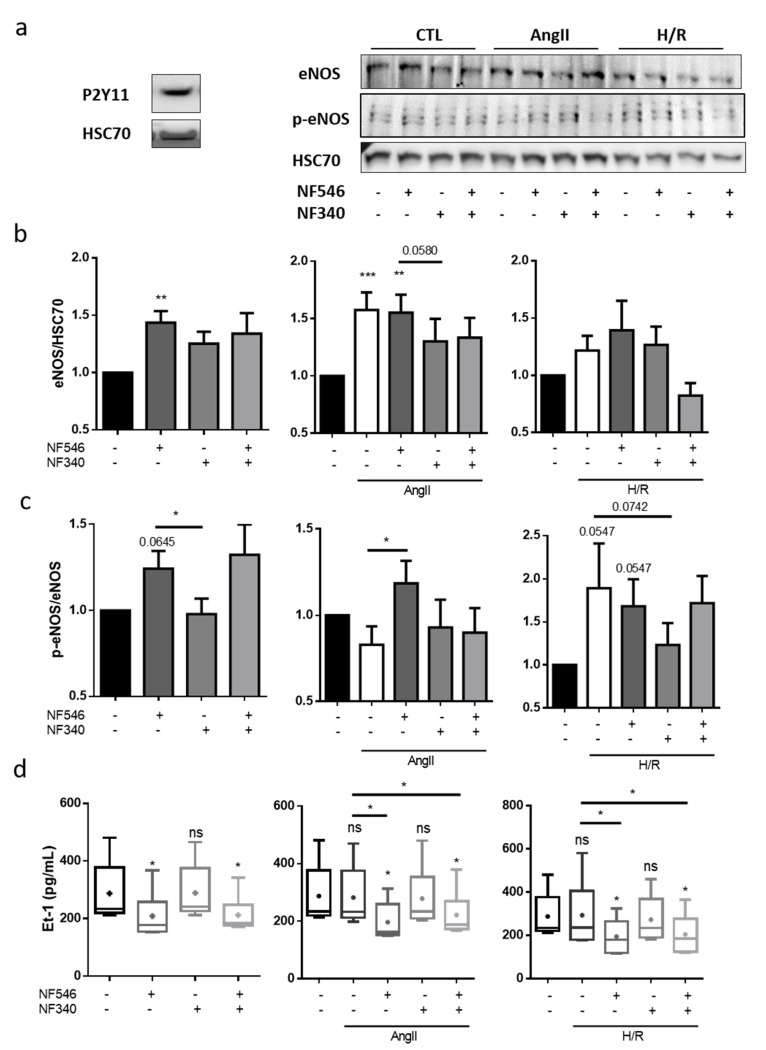
Vascular tone regulation by ECs is modulated by P2Y11 ligands. (**a**) P2Y11 protein expression on HUVECs was analyzed by western blot, showing a band at 50 kDa (n = 3). (**b**,**c**) HUVECs were incubated 30 min with basal medium, AngII (100 nM) or submitted to 5 h hypoxia followed by 30 min reoxygenation (H/R), and incubated with the P2Y11 agonist NF546 (10 µM) or/and antagonist NF340 (10 µM). Endothelial nitric oxyde synthase (eNOS) and p-ser1177-eNOS protein expression were analyzed by western blot. In the CTL group, NF546 increased eNOS expression (n = 13) and p-ser1177-eNOS (n = 10). In the AngII group, eNOS expression was increased in the presence of AngII; AngII+NF546 (n = 13) and p-ser1177-eNOS was increased compared to AngII condition (n = 10); and P2Y11 receptor (P2Y11R) antagonist NF340 tended to decrease eNOS expression compared to AngII condition. In the H/R group, H/R and NF546 tended to increase eNOS expression (n = 13) and increase p-ser1177-eNOS (n = 10). (**d**) HUVECs were incubated 12 h with basal medium, AngII (100 nM) or submitted to 5 h hypoxia followed by 12 h reoxygenation (H/R), and addition of P2Y11R agonist NF546 (10 µM) and/or antagonist NF340 (10 µM). Endothelin-1 release was evaluated in HUVECs supernatant by ELISA assay. AngII or H/R did not show significant changes in endotheline-1 (Et-1) release. In the CTL, AngII and H/R groups, P2Y11R activation by NF546 decreased Et-1 release (n = 6). * p < 0.05, ** p < 0.01 and *** p < 0.01, compared to CTL condition if not indicated. Data given as mean ± SEM and box plot as median and mean + min to max.

We then tried to evaluate how 30 min H/R or AngII incubation could modulate eNOS expression and its activation though the phosphorylation of ser1177 (p-eNOS) in HUVECs. First, we showed that HUVECs expressed P2Y11 receptor (Figure 3a). In the presence of AngII, the eNOS expression was increased at the protein level (from 1 to 1.57 ± 0.15) and tended to be increased after the H/R sequence (from 1 to 1.22 ± 0.12) (Figure 3b). We performed similar experiments with NF546 and NF340 incubations. Under basal condition, P2Y11 agonist increased eNOS (from 1 to 1.43 ± 0.1) and p-eNOS (from 1 to 1.24 ± 0.1) expression. The AngII-induced eNOS expression was decreased by P2Y11R antagonist (from 1.57 ± 0.15 to 1.3 ± 0.19) and P2Y11R agonist increased p-eNOS protein level compared to the AngII condition (from 0.82 ± 0.1 to 1.18 ± 0.13). After H/R, P2Y11R agonist seemed to increase eNOS expression (from 1.22 ± 0.12 to 1.39 ± 0.2) and P2Y11R antagonism seemed to decrease p-eNOS level (from 1.89 ± 0.5 to 1.23 ± 0.2) (Figure 3b,c). H/R seemed to decrease P2Y11R expression in HUVECs (Figure S1b), which could explain the lower modulation effect observed in the H/R groups.

Endothelin-1 (Et-1) is one of the major signaling molecules that induce contraction [17]. After 12 h treatment, AngII and H/R did not modify Et-1 secretion by HUVECs in our model (Figure 3d). NF546 significantly reduced Et-1 secretion in CTL condition (from 287.5 ± 43.2 to 208.7 ± 33.3) as well as in presence of AngII (from 282.3 ± 42.8 to 196.8 ± 27.3) and after H/R (from 292.9 ± 62.5 to 194.3 ± 32.5) (Figure 3d).

### 2.5. P2Y11 Agonist NF546 Decreases H_2_O_2_ Production in Response to Angiotensin II Exposure

**Figure 4:**
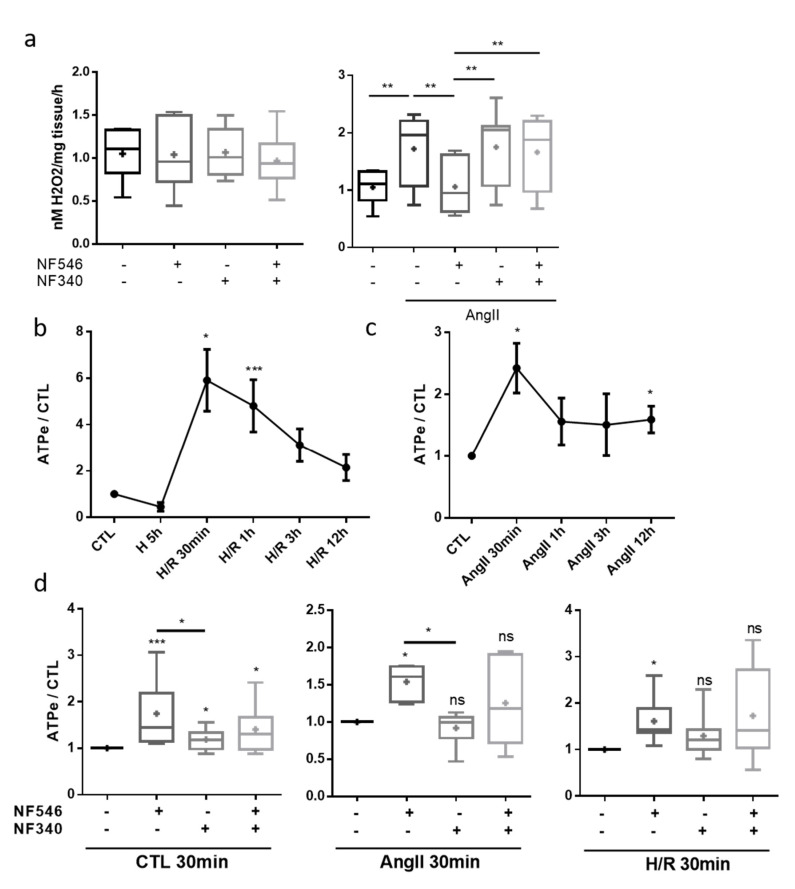
P2Y11 ligands regulate vascular stress factors release. (**a**) H_2_O_2_ release by aortic rings was evaluated by FOX assay. Rings were incubated 30 min in FOX assay reagent with or without AngII (100 nM), NF546 (10 µM) or NF340 (10 µM). P2Y11 ligands NF546 and NF340 had no effect on H_2_O_2_ release under the basal condition. AngII incubation increased H_2_O_2_ release compared to the CTL group. NF546 incubation suppressed the effect of AngII by restoring basal H_2_O_2_ level release (n = 9). (**b**–**d**) Extracellular ATP release was measured in HUVEC supernatants. HUVECs were subjected to 5 h hypoxia and supernatants were collected after several reoxygenation durations (30 min–12 h), or subjected to AngII incubation (30 min–12 h), in presence or absence of NF546 and/or NF340. (**b**) Hypoxia alone tended to decrease eATP secretion. Short times of reoxygenation (30 min–1 h) significantly increased eATP secretion, which progressively returned to the basal level. (**c**) Short exposures to AngII (30 min–1 h) increased eATP secretion, which progressively returned to the basal level. (**d**) After 30 min incubation, NF546 increased eATP secretion in the CTL (n = 11), H/R group (n = 7) and in the AngII group (n = 6). * *p* < 0.05, ** *p* < 0.01 and *** *p* < 0.01, compared to the CTL condition, if not indicated. Data given as mean ± SEM and box plot as median and mean + min to max.

In order to elucidate whether the protective effect of the P2Y11 agonist was linked to changes in ROS production, we performed measurements of H_2_O_2_ production in aorta segments. AngII exposure increased H_2_O_2_ production by the aortic rings compared to the CTL (from 1.05 ± 0.09 to 1.72 ± 0.2). In the latter, NF546 and NF340 had no effect on basal H_2_O_2_ production (Figure 4a). In the AngII-treated group, the P2Y11 agonist decreased H_2_O_2_ release by vessels (from 1.72 ± 0.2 to 1.06 ± 0.15), while the P2Y11 antagonist had no effect. The combination of both NF546 and NF340 treatment had no effect on H_2_O_2_ production (Figure 4a).

### 2.6. Endothelial Cells Release eATP after Stress Exposure

Exposure to hypoxia alone tended to decrease adenosine triphosphate (ATP) release by HUVECs (from 1 to 0.45 ± 0.18). After reoxygenation, we measured significantly higher concentrations of eATP compared to the CTL, with a maximum reached around 30 min (from 1 to 5.9 ± 1.3), followed by a progressive return to basal secretion levels after prolonged reoxygenation (Figure 4b). The same profile was observed after incubation with AngII, with an eATP secretion peak reached around 30 min (from 1 to 2.4 ± 0.4) (Figure 4c). These results suggest a potential autocrine and paracrine effect of eATP on cells subjected to stress conditions. P2Y11 agonist significantly increased eATP secretion in the CTL (from 1 to 1.74 ± 0.21), following the H/R sequence (from 1 to 1.61 ± 0.18) and in AngII condition (from 1 to 1.54 ± 0.09) after 30 min (Figure 4d). 

### 2.7. P2Y11R Activation Decreases SMC Proliferation in Basal and Stress Conditions but Not after Hypoxia 

**Figure 5:**
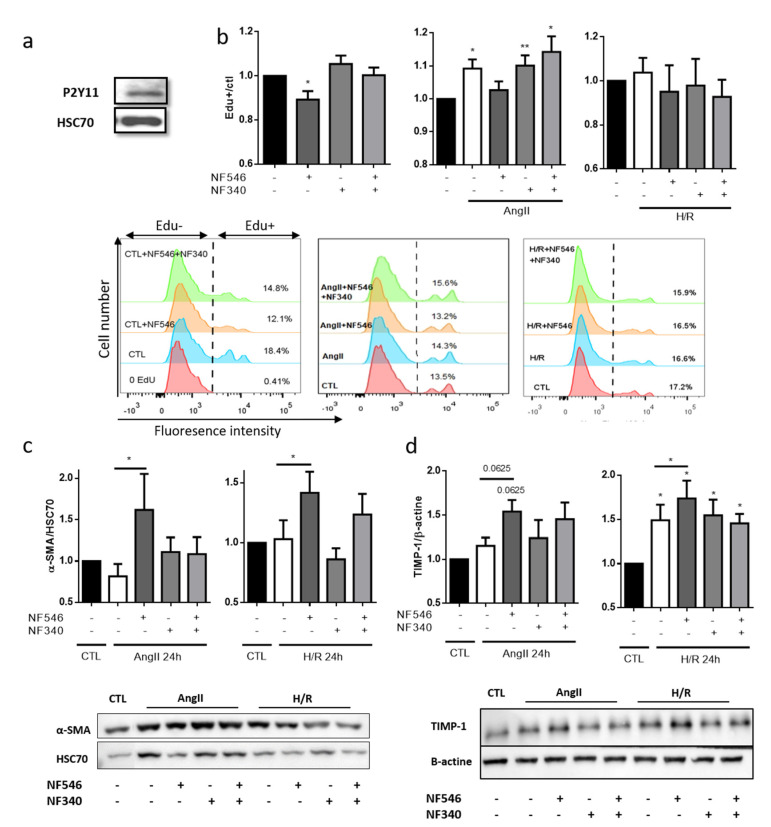
P2Y11R activation reduces SMC switch towards synthetic phenotype. (**a**) P2Y11R expression on HCASMC was analyzed by western blot. We observed a band at 50 kda with P2Y11R antibody. (**b**) HCASMC proliferation was assessed by EdU incorporation during 72 h, and P2Y11R was modulated by NF546 (10 µM) and NF340 (10 µM). Under basal condition, NF546 significantly decreased HCASMC proliferation (n = 9). AngII (100 nM) incubation significantly increased HCASMC proliferation, while NF546 decreased this AngII-induced proliferation (n = 9). Previous exposure to 5 h hypoxia did not modify HCASMC proliferation, and neither NF546 nor NF340 induced differences in proliferation (n = 5). (**c**,**d**) HCASMC phenotype was determined according to the expression of α-SMA and TIMP-1 (contractile) after 24 h, evaluated by western blot. In AngII and H/R groups, NF546 increased α-SMA and TIMP-1 expression (n = 5–8). * *p* < 0.05 and ** *p* < 0.01, compared to CTL condition, if not indicated. Data given as mean ± SEM.

Vascular dysfunction can be characterized by a phenotypical switch of SMC from quiescent contractile to pro-proliferative synthetic phenotype, associated with intimal hyperplasia [18]. We first confirmed that human coronary artery smooth muscle cells (HCASMCs) expressed P2Y11 receptors (Figure 5a). In these cells, in basal conditions, pharmacological activation of P2Y11 with NF546 significantly decreased their proliferation, measured by EdU incorporation, after 72 h (from 1 to 0.89 ± 0.03), whereas P2Y11R inhibition seemed to increase their proliferation (from 1 to 1.05 ± 0.03) (Figure 5b). Under stress condition as induced by AngII, we observed an increase in HCASMC proliferation (from 1 to 1.09 ± 0.02), prevented by NF546 (from 1.09 ± 0.02 to 1.02 ± 0.02). Surprisingly, we noted no significant effect of P2Y11R modulation in the H/R condition (Figure 5b). This could be explained by a downmodulation of P2Y11R expression, which seems to be decreased after H/R (Figure S1a). 

### 2.8. P2Y11 Activation Decreases SMC Phenotype Switch Toward Synthetic Phenotype

The HCASMC phenotype was evaluated according to their expression level of α-SMA and TIMP-1 as markers of contractile phenotype, and vimentin and MMP-2 as markers of synthetic phenotype. P2Y11 agonist NF546 significantly increased α-SMA and TIMP-1 expression after AngII exposure (from 0.81 ± 0.15 to 1.61 ± 0.4 and from 1.15 ± 0.09 to 1.54 ± 0.13) and after H/R (from 1.03 ± 0.15 to 1.41 ± 0.17 and from 1.49 ± 0.17 to 1.73 ± 0.2) (Figure 5c,d). Despite a lower modulation, vimentin expression tended to be increased after AngII (from 1 to 1.27 ± 0.19) and H/R (from 1 to 1.42 ± 0.2) exposure. P2Y11R agonist seemed to decrease vimentin expression only in the AngII group (from 1.27 ± 0.19 to 1.09 ± 0.12), and P2Y11R antagonist tended to increase vimentin expression after H/R exposure (from 1.42 ± 0.2 to 2.5 ± 1.3) (Figure S2a), suggesting that P2Y11R basal activation is sufficient to maintain a moderate vimentin expression. P2Y11R modulation did not seem to modulate MMP-2 expression (Figure S2b). Altogether, these data suggest that P2Y11 agonist modulates SMC phenotype in favor of the contractile one. 

### 2.9. P2Y11 Activation Prevents SMC Proliferation Induced by Endothelial Cells Exposed to HR or AngII

**Figure 6:**
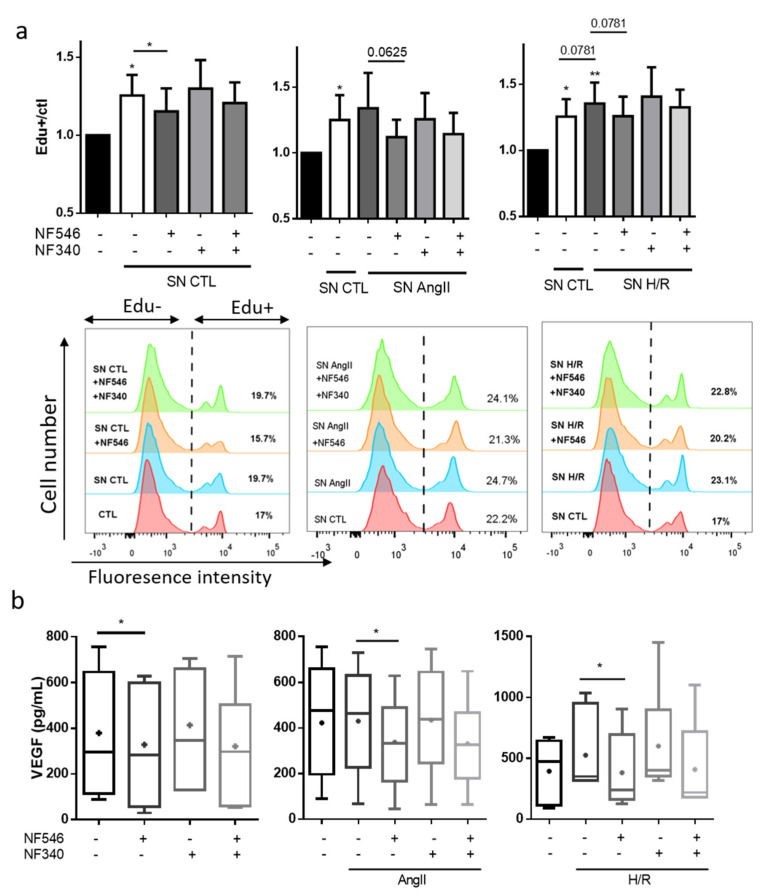
P2Y11R modulates growth factors secretion in the crosstalk between SMC and EC. (**a**) HUVECs were incubated with basal medium, AngII (100 nM) or submitted to 5 h hypoxia followed by 12 h reoxygenation (H/R), and addition of P2Y11R agonist NF546 (10 µM) and antagonist NF340 (10 µM). Supernatant (SN) was harvested after 12 h and incubated with HCASMC and EdU (10 µM) for 24 h. HCASMC proliferation was evaluated by Edu incorporation (n = 7). CTL HUVECs supernatant significantly increased HCASMC proliferation compared to the basal medium. NF546 significantly decreased this pro-proliferative effect. HUVEC AngII-conditioned medium increased HCASMC proliferation compared to the HUVEC basal conditioned medium. NF546 tended to decrease this pro-proliferative effect (*p* = 0.06). HUVEC H/R conditioned medium tended to increase HCASMC proliferation compared to HUVEC basal conditioned medium (*p* = 0.07). NF546 tended to decrease this pro-proliferative effect (*p* = 0.07). (**b**) HCASMC were incubated with basal medium, AngII (100 nM) or submitted to 5 h hypoxia followed by 24 h reoxygenation (H/R), and addition of P2Y11R agonist NF546 (10 µM) and antagonist NF340 (10 µM). VEGF secretion was evaluated in the supernatant by ELISA assay. AngII did not modify VEGF secretion and H/R tended to increase VEGF secretion. VEGF secretion was decreased by NF546 in the CTL (n = 7), AngII (n = 9) and H/R (n = 7) groups. * *p* < 0.05 and ** *p* < 0.01, compared to the CTL condition, if not indicated. Data given as mean ± SEM and box plot as median and mean + min to max.

To assess the role of ECs on the modulation of SMC proliferation, we incubated HCASMC with conditioned media coming from HUVECs under different conditions. We observed that supernatant from HUVECs grown in basal conditions significantly increased EdU incorporation by 25 ± 13% after 24 h. EdU incorporation in HCASMC was also increased when stimulated with supernatants of HUVECs exposed to AngII (from 1.25 to 1.34 ± 0.26) or H/R (from 1.25 ± 0.13 to 1.35 ± 0.15). These effects were prevented by P2Y11 activation in CTL group (from 1.25 ± 0.13 to 1.15 ± 0.14), AngII group (from 1.34 ± 0.26 to 1.12 ± 0.13, *p* = 0.06) and H/R group (from 1.35 ± 0.15 to 1.25 ± 0.14, *p* = 0.07) (Figure 6a).

### 2.10. SMC VEGF Secretion Is Modulated by P2Y11R

To further improve our understanding of the crosstalk between ECs and SMCs, we assessed whether VEGF secretion by SMC was altered under stress conditions. AngII exposure did not increase VEGF secretion. However, VEGF secretion tended to be increased by H/R (from 395 ± 89.2 to 525.9 ± 121). VEGF secretion was decreased by the addition of the P2Y11R agonist NF546, in the CTL (from 378.9 ± 96.7 to 328.4 ± 91.1), AngII (from 430 ± 77 to 336.8 ± 64) and H/R (from 525.9 ± 121 to 382.2 ± 114) groups, and tended to be increased by the P2Y11R antagonist NF340 in the CTL, AngII and H/R groups (Figure 6b). 

## 3. Discussion

In this study, we identified the purinergic G-protein coupled receptor P2Y11 as a potent target to modulate stress-induced vascular dysfunction. We showed that the activation of the P2Y11R-like improved vasomotor response in a rat AngII-induced vascular dysfunction model in an EC-dependent manner. This was modulated by NO and endothelin-1 release, but also by stress factors such as eATP and H_2_O_2_ production. We reported that P2Y11R activation decreased SMC proliferation and phenotype switch in response to stress stimuli. In addition, P2Y11R activation decreased the ECs’ secretome pro-proliferative effect on SMC. We are the first to describe a role of P2Y11R activation in the prevention and treatment of vascular dysfunction, a key process in the pathophysiology of hypertension and myocardial I/R injury. 

In our model of rat thoracic aortic rings, AngII induced vascular dysfunction, by impairing vasocontractile and vasorelaxation responses. The activation of P2Y11R-like by its specific agonist decreased this AngII-induced dysfunction by restoring a basal vasomotor response. We showed that this P2Y11R-like protective effect was lost in the absence of endothelial cells, suggesting that this mechanism was endothelium-dependent. This is in contradiction with the work of Prada et al. who reported that P2Y11R activation increased [Ca^2+^] influx and vasoconstriction in a diabetic hyperglycemia model of vascular myocytes [19]. They reported that this modulation was EC-independent, but their diabetic model was different compared to our vascular dysfunction models. Diabetes is a condition associated with chronic inflammation, which may modify purinergic signaling.

NO is a key molecule in the maintenance of vascular function and relaxation response [20]. It is well known that AngII increases NO production and eNOS expression in endothelial cells [21], which balances its vasoconstrictor effects. At the opposite, H/R reduces both NO production and eNOS expression [22,23]. Kalinowski et al. reported that ATP was a signaling molecule leading to NO-dependent vasodilation, mediated by P2YR and eNOS [24]. These results were confirmed by da Silva et al. who showed on a HUVEC model that ATP (100 µM) induced eNOS ser1177 phosphorylation, mediated by increased intracellular [Ca^2+^] [25]. Here, we identified P2Y11R as one of the possible receptors involved in this previous work. In response to L-NAME, a NOS inhibitor, we observed an increase of contraction in the presence of AngII. Activation of P2Y11R-like normalized this excessive vasocontraction, suggesting that its activation might increase NO bioavailability or decrease the AngII effect. In our HUVEC model, we showed that, after a short incubation period, P2Y11R activation increased eNOS expression. In the presence of AngII, eNOS expression was increased, and this was potentiated by P2Y11R activation and decreased by P2Y11R inhibition. 

Injuries and vascular dysfunction triggered by I/R and AngII are partly due to the generation of ROS in the microenvironment [26]. We showed that AngII induced an increase in H_2_O_2_ production in our aortic rings model. This effect was suppressed by P2Y11R-like activation, further reinforcing its protective effect. Our group already showed that P2Y11R activation decreased mitochondrial oxidative stress in cardiomyocytes though the activation of PKCε [12]. This mechanism could be an explanation to our observation: P2Y11R activation in ECs may enhance PKCε phosphorylation in S729, reducing H_2_O_2_ production and release. Moreover, it is well established that Ca^2+^ level modulates ROS formation [27], which is concordant with the activation of P2Y11R and subsequently the Gq protein. 

Intimal hyperplasia is a leading cause of vessel dysfunction. Vascular cells activation by injury or inflammation induce SMC phenotype switch, proliferation and migration to the intima, altogether contributing to intimal hyperplasia [28]. Here we showed that P2Y11R activation decreased SMC proliferation in basal and stress condition but not after hypoxia. This suggests that P2Y11R can modulate SMC proliferation. This modulation capacity may be decreased after H/R, possibly through a decrease in the expression of P2Y11R, as we observed a decrease in P2Y11R mRNA level after H/R (Figure S1a). Endothelial cells secretory profile is partially responsible for the induction of SMC proliferation [29]. We showed that H/R and AngII increased EC secretome-induced SMC proliferation. P2Y11R activation decreased this EC secretome pro-proliferative effect. Overall, these observations indicated that P2Y11R modulates SMC proliferation capacity both directly and in a paracrine manner, through the EC pro-proliferative secretome. 

Several limitations of our work need to be acknowledged. The angiotensin II model, although not representative of the exact physiologic response to injuries, offers a good model to mimic vascular dysfunction and its involved mechanisms. High concentrations of AngII in the cardiovascular system induce vascular injury and are thus a validated in vitro model of vascular dysfunction. The activation of the renin-angiotensin-aldosterone system is a permanent feature, common to all cardiovascular diseases, from arterial hypertension to chronic heart failure, and as such adds to the relevance of the model. Surprisingly, AngII by itself did not induce thoracic aortic rings contraction, even at high concentrations. Zhou et al. showed differential contraction in response to AngII in murine arteries of different origin. They reported an induction of contraction in response to AngII in abdominal aorta, femoral and carotid artery, but no response in thoracic aorta [30], which could explain our results.

Even though the ortholog gene of P2Y11R has not been found in rodent, several studies highlighted a functional P2Y11R-like, through the same physiological response to its specific P2Y11R agonist and antagonist, namely NF546 and NF340, in human and rodent cells and by showing an increase in intracellular Ca^2+^ concentration and cAMP, in favor of both Gs and Gq proteins activation [31]. Our data match these observations and support the existence of a P2Y11R-like in a rodent’s vessels. This fact and the results presented here would certainly benefit in the future from the development of a mouse genetic model of P2Y11 modulation.

Our results suggest that P2Y11R activation may improve stress-induced vascular dysfunction by maintaining a good balance between pro-constrictors and pro-relaxant factors as well as reducing vascular excessive response to injuries. Considering all the data accumulated on this particular purinergic receptor from our laboratory and others, a strategy based upon the activation of P2Y11R may be a significant therapeutic add-on to all situations where vascular dysfunction plays an important role, for example, in cardiovascular heart disease and solid organ transplantation [32]. Moreover, these findings could be extended to many other vascular diseases as endothelial dysfunction is associated with the development of atherosclerosis and arterial hypertension [33,34]. 

## 4. Materials and Methods 

### 4.1. Aortic Rings Preparation 

All experimental procedures used in this study were conducted in accordance with the Directive 2010/63/EU and the Romanian Law nr. 43/May 2014 regarding the protection of animals used for scientific purposes. The experimental protocol was approved by the Committee for Research Ethics of the “Victor Babeş” University of Medicine and Pharmacy Timişoara, Romania (nr. 11/31 March 2017, extended in 5 February 2019).

Thoracic aortas were obtained from 3-months male Wistar rats. Briefly, rats were anesthetized with a mixture of Ketamine-Xylazine (100 mg/kg–10 mg/kg, i.p.). After thoracotomy, blood was drained and replaced with Hank’s solution (HBSS, AppliChem). Thoracic aorta was removed, and placed in ice-cold Hank’s solution, cleaned from fat, and cut into segments to obtain rings. For endothelial cells-denuded aortic rings studies, the aortic rings were incubated 60 s in 3-[(3-Cholamidopropyl)dimethylammonio]-1-propane-sulfonate (CHAPS) solution (Sigma-Aldrich-Merck, Saint Quentin Fallavier, France) (5 mg/mL CHAPS, 50g/L glucose). 

### 4.2. Vascular Function Analysis

Rat aortic rings were mounted on an isometric force transducer (Myograph DMT 620M) in an organ chamber. This chamber contained 5 mL of Krebs solution equilibrated 30 min at 37 °C with O_2_, and 10 µM diclofenac (Sigma-Aldrich) to block the generation of prostaglandins, then the major vasodilator remaining active is the NO. Aortic rings were then stretched and equilibrated at a tension of 2 g (isometric tension). The maximal contraction level was evaluated in response to a 80 mM KCl solution. After washing steps and equilibration, aortic rings were exposed to angiotensin II (AngII) (100 nM, Sigma-Aldrich) or vehicle (control group) during 30 min. Both the control group and the AngII-treated group were incubated simultaneously with or without NF546 (10 µM, Tocris, R&D System Biotechne, Lille, France), NF340 (10 µM, Tocris, R&D System Biotechne, Lille, France), or both. These are respectively the specific agonist and antagonist of P2Y11R. Contraction was evaluated in response to phenylephrine (Sigma-Aldrich), and was adjusted to reach a preconstriction level of 80% of the contraction elicited by KCl. Relaxation was evaluated in response to cumulative doses of acetylcholine (Sigma-Aldrich-Merck, Saint Quentin Fallavier, France). 

Nitric oxide bioavailability was assessed by evaluation of the vasoconstrictive response to the eNOS inhibitor N(ω)-nitro-L-arginine methyl ester (L-NAME, 10 µM, Sigma-Aldrich). Aortic rings were preconditioned with phenylephrine to reach 10% of the maximal KCl-induced constriction before L-NAME treatment.

### 4.3. Cell Culture and Reagents

Human umbilical vein endothelial cells (HUVECs) and human coronary artery smooth muscle cells (HCASMC) and their corresponding culture media (endothelial cell growth medium 2 and smooth muscle cell growth medium 2) were purchased from Promocell and cultured according to the manufacturer’s instructions. Cells were used from passage 3 to 6 for experimental procedures. 

For H/R experiments, HUVEC and HCASMC were submitted to 5 h hypoxia (1% O_2_, 5% CO_2_, DPBS with CaMg) in a hypoxia chamber (INVIVO_2_ 200, Ruskinn Technology, AWEL internaltional, Blain, France). DPBS was equilibrated overnight before the experiment. Several durations of reoxygenation (DMEM/F12, 5% CO_2_, 21% O_2_) were used depending on the experiment and the type of cell. Hypoxia and reoxygenation durations were based on previous observations from our group [12,13,15]. All modulating agents were added at the onset of the reoxygenation. 

Concomitantly, a simulation of stress environment was induced by AngII (100 nM). Modulation of P2Y11R was performed with its agonist NF546 (10 µM) and antagonist NF340 (10 µM); the maximal concentration effect was chosen according to Meis et al. [35]. 

### 4.4. ROS Measurement

H_2_O_2_-released by aortic rings was evaluated by Ferrous iron xylenol orange Oxidation (FOX) assay using the PeroxiDetect Kit (Sigma-Aldrich-Merck, Saint Quentin Fallavier, France) according to the manufacturer’s instructions. A 30-min incubation was performed in the absence or presence of AngII. The two groups were also incubated at the same time with NF546, NF340 or both.

### 4.5. Secretome Analyses

Extracellular ATP secretion was evaluated in HUVEC supernatant with ATP Bioluminescence Assay Kit HS II (Roche, Sigma-Aldrich -Merck, Saint Quentin Fallavier, France), according to the manufacturer’s instructions. Luminescence was measured with a GloMax^®^-Multi Jr. luminometer (Promega, Charbonniere Les Bains, France). Endothelin-1 release was evaluated by ELISA assay with endotheline-1 (Et-1) ELISA kit (Invitrogen, Thermo Fisher Scientific, Courtaboeuf, France). The supernatant samples were diluted by 10 in order to perform the assay. Vascular Endothelial Growth Factor (VEGF) secretion by SMC was analyzed in HCASMC supernatant by ELISA assay (Invitrogen, Thermo Fisher Scientific, Courtaboeuf, France). The supernatant samples were diluted by 2 in order to perform the assay.

### 4.6. Western Blot

Thoracic aortas rings or whole cells pellet were lysed in RIPA buffer with protease inhibitor cocktail (Sigma-Aldrich—P8340, Sigma-Aldrich -Merck, Saint Quentin Fallavier, France) and phosphatase inhibitors when needed (Halt Phosphatase Inhibitor Cocktail—Thermo Fisher Scientific, Courtaboeuf, France). The protein concentration was determined by BCA assay (Pierce™ BCA Protein Assay Kit, Thermo Fisher Scientific, Courtaboeuf, France). Proteins were separated by 10% SDS-PAGE and transferred on nitrocellulose membrane by semi-dry electroblotting. Non-specific sites were blocked by incubation in Tris-Buffered Saline (TBS)—tween 0.1% (TBST) -BSA 5% for 1 h at room temperature (RT), followed by primary antibody incubation overnight at 4 °C. Membranes were washed with TBST, then incubated with the appropriate secondary antibody coupled with horseradish peroxydase for 1 h at RT and revealed with Amersham™ECL™ Prime Western Blotting Detection Reagent (GE Healthcare, Fisher Scientific, Illkirch, France) and PXi/PXi Touch gel imaging system (Syngene, Fisher Scientific, Illkirch, France). Proteins expression was quantified by densitometry with ImageJ software.

The antibodies used were HSC70 (1:5000, Santa Cruz, Heidelberg Germany), β-actin (1:1000, Santa Cruz, Heidelberg Germany), hP2Y11 (1:500, Alomone, Jerusalem, Israel), hP2Y11-Peptide (1:500, Alomone, Jerusalem, Israel), eNOS (1:1000, abcam, Cambridge, UK), p-ser1177-eNOS (1:1000, abcam, Cambridge, UK) α-SMA (1:1000, Cell Signaling Technology, Ozyme, St Quentin Yvelines, France), TIMP-1 (1:1000, Cell Signaling Technology, Ozyme, St Quentin Yvelines, France), goat anti-mouse and anti-rabbit secondary antibodies (1:10,000, Biorad, Marne La Coquette, France). 

### 4.7. Evaluation of HCASMC Proliferation

HCASMC, placed in 12-wells plates (100,000 cells/well), were incubated with 10 µM EdU in different conditions. After 72 h, cells were harvested and EdU incorporation was evaluated by fluorescence staining (Click-iT™ EdU Alexa Fluor™ 647 Flow Cytometry Assay Kit, Thermo Fisher Scientific, Courtaboeuf, France) using flow cytometry (BD FACS Canto) according to the manufacturer’s instructions. 

For the assay of HUVEC secretome proliferation modulation, we subjected HUVEC to three conditions: basal condition (12 h—DMEM/F12), H/R condition (5 h—hypoxia, 12 h—reoxygenation—DMEM/F12) or AngII (12 h—DMEM/F12—AngII 100 µM). After the 12 h incubation, supernatant was harvested and centrifuged (10 min—1000 g), EdU (10 µM) was added, then supernatants were incubated 24 h with HCASMC in the same conditions as the proliferation assay. Analyses were performed as previously described. 

### 4.8. Statistical Analyses

Data were analyzed using Wilcoxon test. Data analysis of the dose–effect response curves were performed using the extra sum-of-squares *F* test (comparisons of bottom and top values, EC50 and the Hill slope). *p*-values < 0.05 were considered statistically significant. Data given as mean ± SEM and box plot as median and mean + min to max. Data were analyzed using GraphPad Prism (v6.0, https://www.graphpad.com/scientific-software/prism/). 

## Data Availability

All data in this manuscript are available on reasonable request.

## Supplementary Materials

Supplementary materials can be found at https://www.mdpi.com/1422-0067/22/2/855/s1. Figure S1: P2Y11R mRNA evaluation, Figure S2: Modulation of SMC pro-synthetic phenotype markers.

## Abbreviations

AngII	angiotensin II
α-SMA	alpha-smooth muscle actin
AT1	angiotensin II type 1 receptor
ATP	adenosine triphosphate
CTL	control
eATP	extracellular adenosine triphosphate
EC	endothelial cell
eNOS	endothelial nitric oxyde synthase
Et-1	endotheline-1
H/R	hypoxia/reoxygenation
HCASMC	human coronary artery smooth muscle cells
HSC70	heat shock cognate 70 protein
HUVEC	human umbilical vein endothelial cells
I/R	ischemia/reperfusion
L-NAME	N(ω)-nitro-L-arginine methyl ester
LPS	lipopolysaccharide
MMP-2	matrix metalloProteinase 2
NO	nitric oxide
P2Y11R	P2Y11 receptor
TIMP-1	tissue inhibitor of matrix metalloproteinases 1

## References

[1] Wu C.-H., Mohammadmoradi S., Chen J.Z., Sawada H., Daugherty A., Lu H.S. (2018). Renin-Angiotensin System and Cardiovascular Functions. Arter. Thromb. Vasc. Biol..

[2] Montezano A.C., Cat A.N.D., Rios F.J., Touyz R.M. (2014). Angiotensin II and Vascular Injury. Curr. Hypertens. Rep..

[3] Yang B., Li D., Phillips M.I., Mehta P., Mehta J.L. (1998). Myocardial angiotensin II receptor expression and ischemia-reperfusion injury. Vasc. Med..

[4] Eguchi S., Kawai T., Scalia R., Rizzo V. (2018). Understanding Angiotensin II Type 1 Receptor Signaling in Vascular Pathophysiology. Hypertension.

[5] Mu D.N., Dechend R., Fiebeler A., Park J., Schmidt F., Breu V., Mackman N., Luther T., Schneider W., Gulba D. (2000). Angiotensin II (AT 1) Receptor Blockade Reduces Vascular Tissue Factor in Angiotensin II-Induced Cardiac Vasculopathy. Am. J. Pathol..

[6] Hausenloy D.J., Chilian W., Crea F., Davidson S.M., Bencsik P., Garcia-Dorado D., Van Royen N., Schulz R., Heusch G. (2019). The coronary circulation in acute myocardial ischaemia/reperfusion injury: A target for cardioprotection. Cardiovasc. Res..

[7] Zhao Z.-Q., Corvera J.S., Halkos M.E., Kerendi F., Wang N.-P., Guyton R.A., Vinten-Johansen J. (2003). Inhibition of myocardial injury by ischemic postconditioning during reperfusion: Comparison with ischemic preconditioning. Am. J. Physiol. Circ. Physiol..

[8] Zhao J.-L., Yang Y., You S.-J., Cui C.-J., Gao R. (2007). Different effects of postconditioning on myocardial no-reflow in the normal and hypercholesterolemic mini-swines. Microvasc. Res..

[9] Galaup A., Gomez E., Souktani R., Durand M., Cazes A., Monnot C., Teillon J., Le Jan S., Bouleti C., Briois G. (2012). Protection Against Myocardial Infarction and No-Reflow Through Preservation of Vascular Integrity by Angiopoietin-Like 4. Circulation.

[10] Liu C., Mather S., Huang Y., Garland C.J., Yao X. (2004). Extracellular ATP facilitates flow-induced vasodilatation in rat small mesenteric arteries. Am. J. Physiol. Circ. Physiol..

[11] Dănilă M.D., Privistirescu A., Duicu O.M., Rațiu C.D., Angoulvant D., Muntean D.M., Sturza A. (2017). The effect of purinergic signaling via the P2Y11 receptor on vascular function in a rat model of acute inflammation. Mol. Cell. Biochem..

[12] Benoist L., Chadet S., Genet T., Lefort C., Heraud A., Danila M.D., Muntean D.M., Baron C., Angoulvant D., Babuty D. (2019). Stimulation of P2Y11 receptor protects human cardiomyocytes against Hypoxia/Reoxygenation injury and involves PKCε signaling pathway. Sci. Rep..

[13] Lefort C., Benoist L., Chadet S., Piollet M., Heraud A., Babuty M., Baron C., Ivanes F., Angoulvant D. (2018). Stimulation of P2Y11 receptor modulates cardiac fibroblasts secretome toward immunomodulatory and protective roles after Hypoxia/Reoxygenation injury. J. Mol. Cell. Cardiol..

[14] Bourguignon T., Benoist L., Chadet S., Miquelestorena-Standley E., Fromont G., Ivanes F., Angoulvant D. (2019). Stimulation of murine P2Y11-like purinoreceptor protects against hypoxia/reoxygenation injury and decreases heart graft rejection lesions. J. Thorac. Cardiovasc. Surg..

[15] Chadet S., Ivanes F., Benoist L., Gandonnière C.S., Guibon R., Velge-Roussel F., Babuty M., Baron C., Roger S., Angoulvant D. (2015). Hypoxia/Reoxygenation Inhibits P2Y11 Receptor Expression and Its Immunosuppressive Activity in Human Dendritic Cells. J. Immunol..

[16] Balogh J., Wihlborg A.-K., Isackson H., Joshi B.V., Jacobson K.A., Arner A., Erlinge D. (2005). Phospholipase C and cAMP-dependent positive inotropic effects of ATP in mouse cardiomyocytes via P2Y-like receptors. J. Mol. Cell. Cardiol..

[17] Böhm F., Pernow J. (2007). The importance of endothelin-1 for vascular dysfunction in cardiovascular disease. Cardiovasc. Res..

[18] Geraghty J.G., Stoltenberg R.L., Sollinger H.W., Hullett D.A. (1996). Vascular Smooth Muscle Cells And Neointimal Hyperplasia In Chronic Transplant Rejection. Transplantation.

[19] Prada M.P., Syed A.U., Buonarati O.R., Reddy G.R., Nystoriak M.A., Ghosh D., Simó S., Sato D., Sasse K.C., Ward S.M. (2019). A Gs-coupled purinergic receptor boosts Ca2+ influx and vascular contractility during diabetic hyperglycemia. eLife.

[20] Tousoulis D., Kampoli A.-M., Papageorgiou C.T.N., Stefanadis C. (2012). The Role of Nitric Oxide on Endothelial Function. Curr. Vasc. Pharmacol..

[21] Suzuki H., Eguchi K., Ohtsu H., Higuchi S., Dhobale S., Frank G.D., Motley E.D., Eguchi S. (2006). Activation of Endothelial Nitric Oxide Synthase by the Angiotensin II Type 1 Receptor. Endocrinology.

[22] Huang J., He G.-W., Xue H.-M., Yao X., Liu X.-C., Underwood M.J., Yang Q. (2011). TRPC3 channel contributes to nitric oxide release: Significance during normoxia and hypoxia–reoxygenation. Cardiovasc. Res..

[23] Zhu M., Ding J., Jiang H., Kong L., Sun Z., Chen J.-W., Miao C. (2015). Propofol ameliorates endothelial inflammation induced by hypoxia/reoxygenation in human umbilical vein endothelial cells: Role of phosphatase A2. Vasc. Pharmacol..

[24] Kalinowski L., Dobrucki L.W., Szczepanska-Konkel M., Jankowski M., Martyniec L., Angielski S., Malinski T. (2003). Third-Generation β-Blockers Stimulate Nitric Oxide Release From Endothelial Cells Through ATP Efflux. Circulation.

[25] Da Silva C.G., Specht A., Wegiel B., Ferran C., Kaczmarek E. (2009). Mechanism of Purinergic Activation of Endothelial Nitric Oxide Synthase in Endothelial Cells. Circulation.

[26] Cai H., Harrison D.G. (2000). Endothelial Dysfunction in Cardiovascular Diseases: The Role of Oxidant Stress. Circ. Res..

[27] Görlach A., Bertram K., Hudecova S., Krizanova O. (2015). Calcium and ROS: A mutual interplay. Redox Biol..

[28] Mitchell R.N., Libby P. (2007). Vascular Remodeling in Transplant Vasculopathy. Circ. Res..

[29] Waybill P.N., Chinchilli V.M., Ballermann B.J. (1997). Smooth Muscle Cell Proliferation in Response to Co-culture with Venous and Arterial Endothelial Cells. J. Vasc. Interv. Radiol..

[30] Zhou Y., Dirksen W.P., Babu G.J., Periasamy M. (2003). Differential vasoconstrictions induced by angiotensin II: Role of AT1 and AT2 receptors in isolated C57BL/6J mouse blood vessels. Am. J. Physiol. Circ. Physiol..

[31] Kennedy C. (2017). P2Y11 Receptors: Properties, Distribution and Functions. Cannabinoids Neuropsychiatr. Disord..

[32] Dănilă M.-D., Piollet M., Aburel O.-M., Angoulvant D., Lefort C., Chadet S., Roger S., Muntean D.M., Ivanes F. (2020). Modulation of P2Y11-related purinergic signaling in inflammation and cardio-metabolic diseases. Eur. J. Pharmacol..

[33] Xie N., Wang C., Lian Y., Wu C., Zhang H., Zhang Q. (2014). Inhibition of mitochondrial fission attenuates Aβ-induced microglia apoptosis. Neuroscience.

[34] Brandes R.P. (2014). Endothelial Dysfunction and Hypertension. Hypertension.

[35] Meis S., Hamacher A., Hongwiset D., Marzian C., Wiese M., Eckstein N., Royer H.-D., Communi D., Boeynaems J.-M., Hausmann R. (2009). NF546 [4,4′-(Carbonylbis(imino-3,1-phenylene-carbonylimino-3,1-(4-methyl-phenylene)-carbonylimino))-bis(1,3-xylene-α,α′-diphosphonic Acid) Tetrasodium Salt] Is a Non-Nucleotide P2Y11 Agonist and Stimulates Release of Interleukin-8 from Human Monocyte-Derived Dendritic Cells. J. Pharmacol. Exp. Ther..

