# Supplementary material for: P2Y11 Agonism Prevents Hypoxia/Reoxygenation- and Angiotensin II-Induced Vascular Dysfunction and Intimal Hyperplasia Development
Source: Int J Mol Sci. 2021 Jan 16;22(2):855. doi: 10.3390/ijms22020855 (PMC7829863; doi:10.3390/ijms22020855)

Supplemental datas

Materials and methods

Real Time qPCR

HCASMC were harvested in dry pellet then mRNA were extracted with NucléoSpin® RNA Kit (Machery Nagel) according to the manufacturer instructions, then transcript in cDNA with PrimeScript™RT Reagent kit (Takara). RTqPCR was performed with TB Green™ Premix Ex Taq™ (Takara). The amplification and fluorescence evaluation were realized with the CFX Cennect™ Real Time System (Bio-Rad). F-P2Y11: TAGCAGACACAGGCTGAGGA, R-P2Y11: CACCAGGAACTCAACCACCA, F-β2-Microglobuline: CCCAAGATAGTTAAGTGGGATCG, R- β2-Microglobuline: TCATCCAATCCAAATGCGGC

Westernblot

Westernblots for vimentine and MMP-2 were performed as describe in the original manuscript. Antibody used were: Vimentin (1:1000, Cell Signaling Technology), MMP-2 (1:1000, Cell Signaling Technology).

Figures

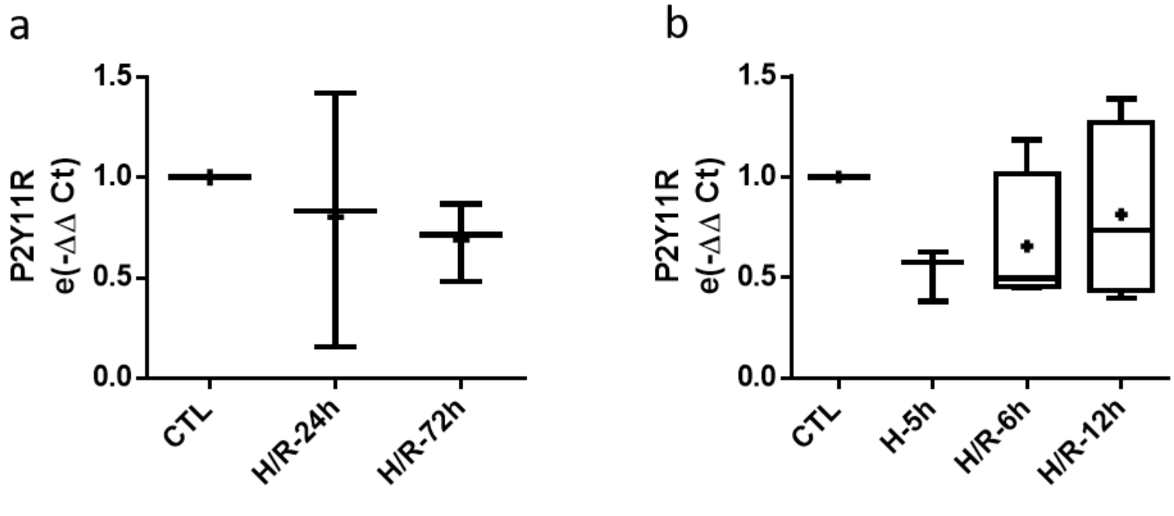

Figure S1: P2Y11R mRNA evaluation

a: Evaluation of P2Y11R mRNA level in HCASMC after 24h or 72h of H/R (n=3). **b**: Evaluation of P2Y11R mRNA level in HUVEC after 5h hypoxia (n=3), or 6h or 12h of H/R (n=4).

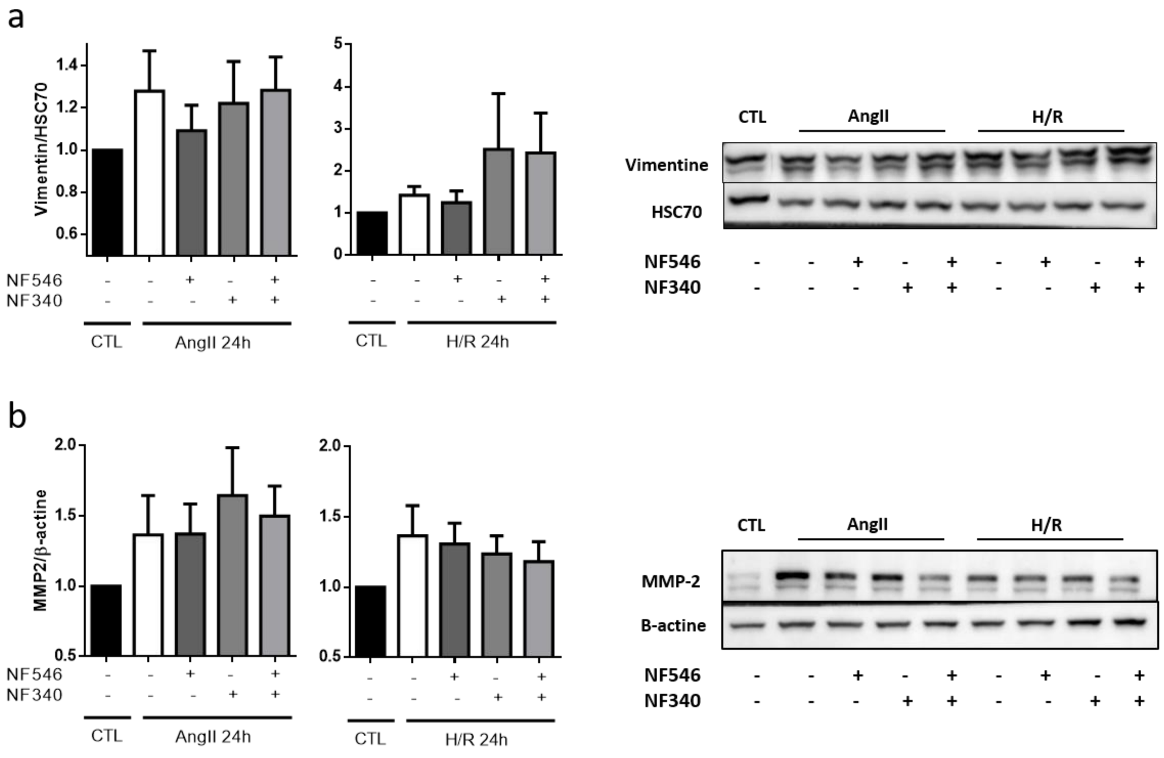

Figure S2: Modulation of SMC pro-synthetic phenotype markers.

HCASMC phenotype was determined according to the expression of vimentin and MMP-2 (synthetic) after 24h, evaluated by western blot. (**a,b**) AngII tended to increase vimentin and MMP-2 expression, NF546 tended to decrease vimentin and NF340 to increase MMP-2 expression. In H/R group, P2Y11R antagonism tended to increase vimentin expression (n=5-8).

Figure S2 (b)

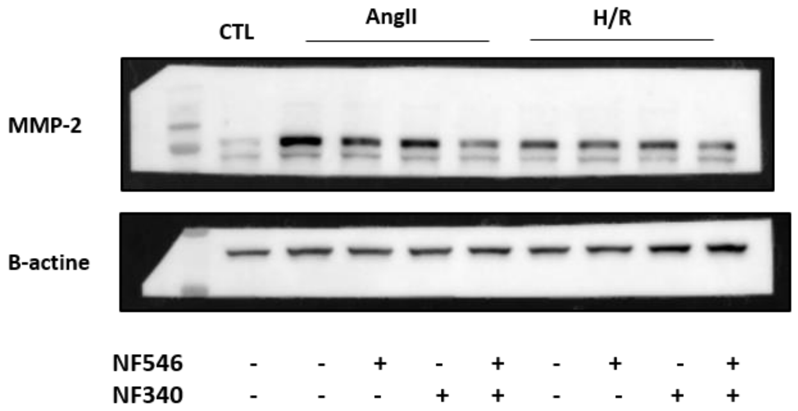

Supplement: Supplementary file 1 [file ijms-22-00855-s001.pdf]
